# Log odds of positive lymph nodes (LODDS)-based novel nomogram for survival estimation in patients with invasive micropapillary carcinoma of the breast

**DOI:** 10.1186/s12874-024-02218-1

**Published:** 2024-04-18

**Authors:** Xiangdi Meng, Furong Hao, Nan Wang, Peiyan Qin, Zhuojun Ju, Daqing Sun

**Affiliations:** 1 Department of Radiation Oncology, Weifang People’s Hospital, No. 151 Guangwen Street, Kuiwen District, Weifang, 261041 Shandong China; 2 Graduate School of Medicine, Gunma University, Maebashi, Japan

**Keywords:** Invasive micropapillary carcinoma, Log odds of positive lymph nodes, Breast cancer-specific survival, Nomogram, Prognosis

## Abstract

**Background:**

Invasive micropapillary carcinoma (IMPC) of the breast is known for its high propensity for lymph node (LN) invasion. Inadequate LN dissection may compromise the precision of prognostic assessments. This study introduces a log odds of positive lymph nodes (LODDS) method to address this issue and develops a novel LODDS-based nomogram to provide accurate prognostic information.

**Methods:**

The study analyzed data from 1,901 patients with breast IMPC from the Surveillance, Epidemiology, and End Results database. It assessed the relationships between LODDS and the number of excised LN (eLN), positive LN (pLN), and the pLN ratio (pLNR), identifying an optimal threshold value using a restricted cubic spline method. Predictive factors were identified by the Cox least absolute shrinkage and selection operator (Cox-LASSO) regression and validated through multivariate Cox regression to construct a nomogram. The model's accuracy, discrimination, and utility were assessed. The study also explored the consequences of excluding LODDS from the nomogram and compared its effectiveness with the tumor-node-metastasis (TNM) staging system.

**Results:**

LODDS improved N status classification by identifying heterogeneity in patients with pLN ratios of 0% (pLN =0) or 100% (pLN =eLN) and setting -1.08 as the ideal cutoff. Five independent prognostic factors for breast cancer-specific survival (BCSS) were identified: tumor size, N status, LODDS, progesterone receptor status, and histological grade. The LODDS-based nomogram achieved a strong concordance index of 0.802 (95% CI: 0.741-0.863), surpassing both the version without LODDS and the conventional TNM staging in all tests.

**Conclusions:**

For breast IMPC, LODDS served as an independent prognostic factor, its effectiveness unaffected by the anatomical LN count, enhancing the accuracy of N staging. The LODDS-based nomogram showed promise in offering more personalized prognostic information.

**Supplementary Information:**

The online version contains supplementary material available at 10.1186/s12874-024-02218-1.

## Background

Invasive micropapillary carcinoma (IMPC) represents a rare but highly invasive breast cancer subtype, accounting for 1% to 10% of all diagnosed cases [1–3]. Unlike invasive ductal carcinoma (IDC), IMPC usually presents with extensive lymph node metastases at diagnosis [4–10]. Specifically, breast IMPC cases typically exhibited a positive lymph node ratio (pLNR) ranging from 44% to 96% [4, 6, 8, 11] and were more likely to have over four positive lymph nodes (pLNs) compared to IDC [7]. Despite many researchers advocating for the examination of as many lymph nodes as possible, a standardized consensus has not been achieved [4, 6, 12]. Therefore, exploring efficient lymph node assessment strategies is crucial for enabling accurate and individualized prognostication and treatment for breast IMPC.

Traditional lymph node staging of breast cancer focuses on the number of pLNs without considering the adequacy of excised lymph nodes (eLNs). This oversight could lead to staging migration and imprecise prognostic assessments [13], especially given the significant tendency of breast IMPC to invade lymph nodes. The log odds of positive lymph nodes (LODDS) — calculated by the ratio of pLNs to negative lymph nodes (nLNs) —has emerged as a potentially important complement, or even an alternative, to traditional N staging [14–17]. However, no studies have established an optimal threshold for LODDS in breast IMPC or assessed its prognostic accuracy.

The American Joint Committee on Cancer (AJCC) tumor-node-metastasis (TNM) staging system has provided valuable, albeit incomplete, prognostic information for breast IMPC. This is because even patients within the same stage can be heterogeneous, potentially limiting the ability for personalized prognostic assessments. To tackle this issue, a nomogram, a visualization of a predictive statistical model, was developed. It considered several key predictors to quantify risk and improve predictive accuracy [18, 19], thus providing a more accurate prognostic estimate than N staging, which only considered the number of pLNs. In addition, the nomogram was reportedly prevalent in various cancer studies and was considered superior to the TNM system alone [20, 21]. Assuming LODDS acts as an independent prognostic factor for breast IMPC, its inclusion in a nomogram could enhance both performance and clinical utility.

In this study, we aimed to evaluate the predictive value of the LODDS for breast IMPC, determine its optimal threshold, and develop a LODDS-based nomogram for predicting breast cancer-specific survival (BCSS). This model was expected to provide higher predictive performance to address the limitation of unclear classification of traditional N staging.

## Material and methods

### Data sources, patient selection and variables

The study was conducted in accordance with the principles of the Declaration of Helsinki. After obtaining access authorization, we retrospectively analyzed the patients diagnosed with breast IMPC [International Classification of Diseases for Oncology (ICD-O-3) code: 8507/3] from the Surveillance, Epidemiology, and End Results (SEER) database between 2010 and 2019 (*n* =2,414). Since the data were obtained from the SEER database, the need for approval from our institutional ethics committee and the requirement for individual patient informed consent were exempted.

Patients were screened for the following exclusion criteria: 1) age <18 years; 2) having distant metastases at diagnosis; 3) absence of histopathological confirmation; 4) diagnosis of non-first primary cancers; 5) less than 1 month of follow-up; 6) ack of crucial clinicopathologic information.

The study included variables such as age at diagnosis, race, marital status, T status, N status, the count of eLNs, the count of pLNs, histologic grade, estrogen receptor (ER) status, progesterone receptor (PR) status, human epidermal growth factor receptor-2 (Her-2) status, chemotherapy, radiation therapy, and surgery. The clinical endpoint was breast cancer-specific survival, with the follow-up period extending from the diagnosis date to the date of death attributable to breast cancer, the last follow-up date, or the predetermined cut-off date.

## Statistical analysis

Clinicopathologic characteristics were presented as counts and percentages for categorical variables and as the means with standard deviations for continuous variables. The chi-square test was used to analyze categorical variables, and the rank-sum test was used for comparing continuous variables. The BCSS was estimated using the Kaplan-Meier method, and differences between subgroups were assessed using the log-rank test.

LODDS was defined as the logarithm of the ratio of the number of pLNs to the number of nLNs. The formula was: ${\text{LODDS}}={\text{log}}(\frac{No. PLNs+0.05}{No. NLNs+0.05})$. To avoid undefined or infinite values when the number of pLNs or nLNs was zero, we added 0.05 to both the numerator and the denominator. We analyzed the distributions of LODDS to eLN, pLN and the positive lymph node ratio (pLNR), respectively. A restricted cubic spline (RCS) method was used to capture the potential non-linear effects of continuous changes in LODDS and their impact on BCSS. Subsequently, the LODDS values corresponding to a hazard ratio (HR) of 1 was identified as the critical threshold. Survival differences between the two subgroups were then assessed using Kaplan Meier curves.

We identified predictive factors using the Cox least absolute shrinkage and selection operator (Cox-LASSO) regression with 5-fold cross-validation to prevent the model from overfitting or underfitting. This method effectively combines the Cox proportional hazards model with LASSO's regularization capabilities, allowing for variables' precise selection and shrinkage, thereby enhancing the model's predictive accuracy and robustness in survival analysis [22, 23]. The selected factors were then incorporated into a multivariate Cox regression analysis to ascertain their independent prognostic relevance for BCSS. From this analysis, we constructed a nomogram.

To measure the accuracy, clinical utility, and discriminatory capability of the model, we employed a calibration plot, decision curve analysis (DCA), and time-dependent area under the curve (AUC), respectively. During internal validation, the time-dependent AUC was re-evaluated by 50 times 10-fold cross-validation, underscoring the sustained reliability of the nomogram over time. To further assess the significance of the LODDS in the model, we compared the Harrell concordance index (C-index), Akaike information criterion (AIC), net reclassification improvement (NRI), and integrated discrimination improvement (IDI) of the nomogram with and without LODDS and compared them with TNM staging.

Statistical analyses were conducted using R (version 4.2.2; http://www.r-project.org). A p value less than 0.05 was considered to indicate statistical significance in this study. The R packages used in the study were as follows: "tableone" package for descriptive statistical analysis; "survival" and "survminer" packages for estimating survival; "rms" package for RCS analysis and nomogram; "glmnet" and "care" packages for Cox-LASSO regression; "ggDCA" package for DCA analysis; the "riskRegression" package for calculating AUC; the "survIDINRI" package for calculating IDI and NRI; the "gghalves", "ggprism" and "ggsci" packages for graph plotting:

## Results

After screening (Additional file 1), 1,901 patients with breast IMPC were included in this study. The majority of these patients were young and middle-aged women (aged ≤70 years, *n* =1440, 75.7%), 78.1% were white people, 10.2% were black people, and 10.2% were of other ethnicities (American Indian/AK Native, Asian/Pacific Islander). In this cohort, 44.6% (848/1901) of breast IMPC patients presented with pLN, and higher-grade N staging was associated with increased mortality (*P* <0.001). In addition, factors such as larger tumor size, higher histologic grade, hormone receptor status, marital status, type of surgery, and chemotherapy were significantly correlated with death from breast IMPC (*P* <0.05). The detailed characteristics were shown in Table 1.

**Table 1:** Clinicopathologic features of the breast cancer patients (*n* =1901)

Characteristics	Whole cohort	Alive	Died of breast cancer	*P value*
	*N* = 1901 (%)	*N* = 1828 (%)	*N* = 73 (%)	
Age				0.290
≤70	1440 (75.7)	1389 (76.0)	51 (69.9)	
>70	461 (24.3)	439 (24.0)	22 (30.1)	
Race				0.206
White people	1484 (78.1)	1433 (78.4)	51 (69.9)	
Black people	224 (11.8)	213 (11.7)	11 (15.1)	
Other people	193 (10.2)	182 (10.0)	11 (15.1)	
Marital status				0.003
Unmarried	792 (41.7)	749 (41.0)	43 (58.9)	
Married	1109 (58.3)	1079 (59.0)	30 (41.1)	
Tumor size				<0.001
≤20 mm	1137 (59.8)	1115 (61.0)	22 (30.1)	
21-50 mm	589 (31.0)	556 (30.4)	33 (45.2)	
>50 mm	175 (9.2)	157 (8.6)	18 (24.7)	
N status				<0.001
N0	1053 (55.4)	1030 (56.3)	23 (31.5)	
N1	547 (28.8)	529 (28.9)	18 (24.7)	
N2	187 (9.8)	174 (9.5)	13 (17.8)	
N3	114 (6.0)	95 (5.2)	19 (26.0)	
eLN, mean (SD)	6.6 (7.2)	6.4 (7.1)	10.6 (8.1)	<0.001
pLN, mean (SD)	1.9 (4.0)	1.8 (3.7)	5.6 (7.2)	<0.001
TNM staging (AJCC-8th)				<0.001
I	819 (43.1)	804 (44.0)	15 (20.5)	
II	733 (38.6)	707 (38.7)	26 (35.6)	
III	349 (18.4)	317 (17.3)	32 (43.8)	
Histological grade				<0.001
I-II	1239 (65.2)	1217 (66.6)	22 (30.1)	
III-IV	662 (34.8)	611 (33.4)	51 (69.9)	
Estrogen receptor status				<0.001
Negative	146 (7.7)	125 (6.8)	21 (28.8)	
Positive	1755 (92.3)	1703 (93.2)	52 (71.2)	
Progesterone receptor status				<0.001
Negative	317 (16.7)	283 (15.5)	34 (46.6)	
Positive	1584 (83.3)	1545 (84.5)	39 (53.4)	
Her-2 status				0.290
Negative	1508 (79.3)	1446 (79.1)	62 (84.9)	
Positive	393 (20.7)	382 (20.9)	11 (15.1)	
Molecular subtypes				<0.001
Luminal A	1444 (76.0)	1397 (76.4)	47 (64.4)	
Luminal B	319 (16.8)	312 (17.1)	7 (9.6)	
Her-2 overexpression	74 (3.9)	70 (3.8)	4 (5.5)	
Triple negative	64 (3.4)	49 (2.7)	15 (20.5)	
Surgery				<0.001
Breast conserving surgery	1078 (56.7)	1052 (57.5)	26 (35.6)	
Mastectomy	823 (43.3)	776 (42.5)	47 (64.4)	
Radiotherapy				0.389
No\Unknown	780 (41.0)	746 (40.8)	34 (46.6)	
Yes	1121 (59.0)	1082 (59.2)	39 (53.4)	
Chemotherapy				0.028
No\Unknown	981 (51.6)	953 (52.1)	28 (38.4)	
Yes	920 (48.4)	875 (47.9)	45 (61.6)	

***Abbreviations:**** eLN* excised lymph node, *pLN* positive lymph node, *SD* Standard deviation, *TNM* Tumor-node-metastasis, *AJCC* American Joint Committee on Cancer, *Her-2* Human epidermal growth factor receptor-2

**Fig. 1:**
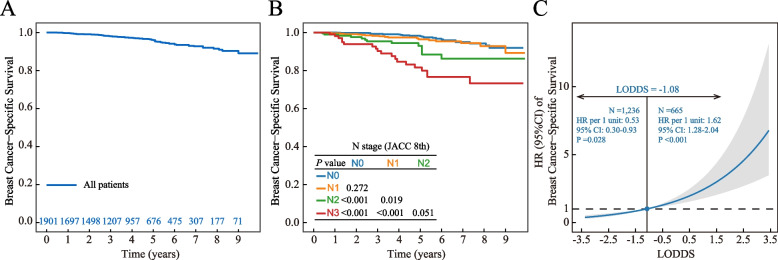
Breast cancer-specific survival (BCSS) for all patients (**A**) and those with different N statuses (**B**), and the hazard ratio (HR) of BCSS changes with lymph node positivity odds (LODDS) (**C**). Abbreviations: AJCC, American Joint Committee on Cancer

The median follow-up period in this study was 48 months, with an interquartile range (IQR) of 27 to 71 months. The 5-year and 8-year BCSS rates for patients were 96.0% [95% confidence interval (CI): 94.8%-97.1%] and 91.5% (95% CI: 89.2%-93.8%), respectively (Fig. 1A). The Kaplan-Meier curves did not show significant differences in survival between patients classified as N0 and N1 (*P* = 0.272) or between those classified as N2 and N3 (*P* = 0.051) (Fig. 1B). RCS analysis revealed a non-linear relationship between the LODDS and BCSS; the LODDS value corresponding to an HR of 1 was calculated (Fig. 1C). When the LODDS was ≤ -1.08, the risk of BCSS decreased by 47% for each unit decrease in LODDS (*P* = 0.028). In contrast, for LODDS values exceeding -1.08, each unit increase in LODDS was associated with a 62% increase in the risk of BCSS (*P*<0.001).

**Fig. 2:**
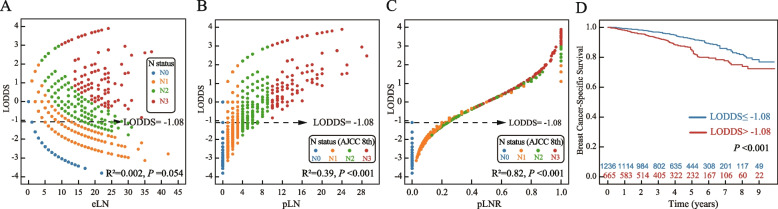
Correlation analysis of log odds of positive lymph nodes (LODDS) with the excised lymph node (eLN) (**A**), the positive lymph node (pLN) (**B**), and the positive lymph node ratio (pLNR) (**C**), and survival analysis of patients in the two subgroups after grouping LODDS based on the optimal cutoff value (D). Abbreviations**:** AJCC, American Joint Committee on Cancer

The study also examined the correlations between LODDS and the number of eLN, pLN, and pLNR. There was no significant correlation between LODDS and eLN (Fig. 2A, R^2^ = 0.002, *P* = 0.054), while pLN exhibited a correlation with LODDS (Fig. 2B, R^2^ =0.39, *P* <0.001). In Figure 2C, most patients in stages N0 and N1, and some in N3, were clustered in the LODDS ≤ -1.08 subgroup, suggesting that LODDS could provide a finer stratification within the same N stage. Moreover, there was a strong correlation between pLNR and LODDS (R^2^ = 0.82, *P*<0.001), indicating that LODDS could effectively stratify patients, especially in cases where the pLNR was 0% or 100% (i.e., when pLN was 0 or equal to eLN (Fig. 2C). Patients were divided into two groups based on a LODDS cutoff value of -1.08. The Kaplan-Meier curve demonstrated superior survival for the group with LODDS ≤ -1.08 compared to the group with LODDS > -1.08 (*P*<0.001, Fig. 2D).

**Fig. 3:**
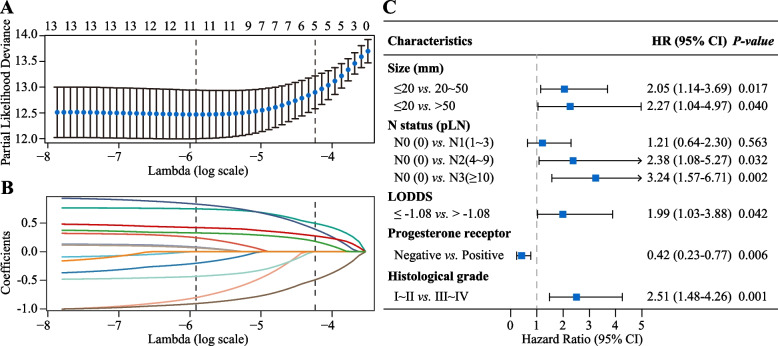
The Cox least absolute shrinkage and selection operator (Cox-LASSO) regression with 5-fold cross-validation (**A** and **B**) and multivariate Cox regression (**C**) for screening and validation of predictors. Abbreviations: pLN, positive lymph node; CI, confidence interval; HR, hazard ratio

**Fig. 4:**
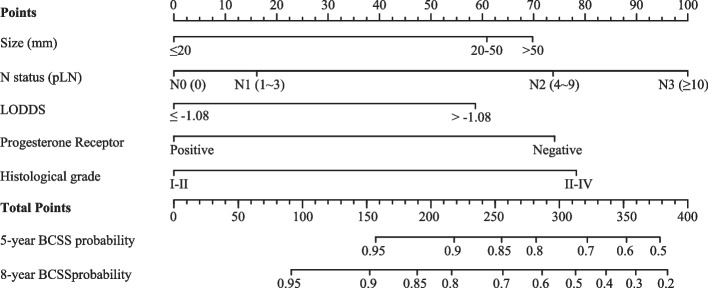
Lymph node positivity odds (LODDS)-based nomogram for predicting breast cancer-specific survival (BCSS) in patients with breast invasive micropapillary carcinoma (IMPC). Abbreviations: pLN, positive lymph node

Predictive factors were selected using the Cox-LASSO with 5-fold cross-validation revealed that a model comprising five factors (tumor size, N status, LODDS, ER status, and histologic grade) performed optimally (Fig. 3A and B). We then validated these five factors as independent prognostic indicators for breast IMPC via multivariate Cox regression (Fig. 3C, all *P* <0.05) and used them to construct a nomogram. This model quantified the risk of all predictive factors as risk scores. By inputting patient information, we obtained 5-year and 8-year BCSS probabilities corresponding to the total risk score (Fig. 4).

**Fig. 5:**
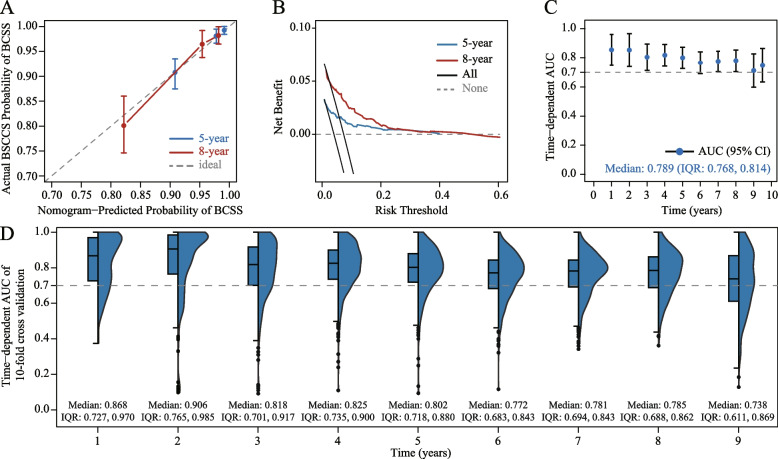
Calibration plots (**A**), decision curve analysis (DCA) (**B**), and time-dependent area under the curve (AUC) curves (**C**) for model predictive accuracy, usefulness, and discrimination, respectively, and the time-dependent AUC values validated by 50 times 10-fold cross-internal validation

The accuracy, clinical utility, and discrimination capabilities of the nomogram were assessed. The calibration curve showed strong agreement between the nomogram's predictions and the actual observations, with both the 5-year and 8-year curves closely aligning with the ideal 45° line (Fig. 5A). The DCA suggested that utilizing the LODDS-based nomogram for guiding medical interventions would provide a greater net benefit compared to blanket treatment strategies, either treating all patients or none (Fig. 5B). The time-dependent AUC analysis demonstrated that the nomogram's ability to discriminate did not decline over time, maintaining commendable stability with a median AUC value of 0.789 (IQR: 0.768-0.814) (Fig. 5C), within a ten-year timeframe. Moreover, the nomogram's performance was validated through internal cross-validation conducted 500 times, revealing consistently high AUC values over the years, with median values ranging from 0.738 to 0.906 (Fig. 5D).

When comparing the performance of the LODDS-based nomogram with that of the non-LODDS version and the TNM staging system (Additional file 2), it was observed that the LODDS-based nomogram had a higher C-index and time-dependent AUC. A smaller AIC indicated a better fit of the model; the removal of LODDS from the nomogram resulted in an increase in the AIC from 905.169 to 910.066, while the AIC for the TNM staging system was higher at 958.694. NRI analysis showed that omitting LODDS from the nomogram decreased its classification accuracy by 19.8% and 10.0% for the 5-year and 8-year NRI, respectively, with all *P* <0.001. Transitioning to the TNM staging system resulted in an even more significant decrease in performance [5-year NRI = –19.9%, *P* <0.001; 8-year NRI = –15.2%, *P* <0.001]. According to the IDI, which evaluates differences in prediction probabilities, the exclusion of LODDS from the nomogram led to a decrease in predictive performance by 2.8% and 2.4% for the 5-year and 8-year IDI, respectively, with all P-values <0.001. The predictive performance of the TNM staging system also experienced a significant decline compared to the LODDS-based nomogram (5-year IDI = –4.6%, *P* <0.001; 8-year IDI = –5.2%, *P* <0.001).

## Discussion

In light of the pronounced lymph node invasive nature of breast IMPC, this study sought a new predictive marker - LODDS, which was identified as an independent prognostic factor for breast IMPC. Crucially, a nomogram incorporating both LODDS and N staging was developed. The advantage of this model is its capacity to preserve accuracy that might be compromised due to staging migration resulting from inadequate lymph node dissection. Furthermore, this nomogram demonstrated superior predictive performance over the TNM staging, offering promising avenues for personalized treatment planning.

Pathologic N staging of breast IMPC is determined by the number of pLNs detected, regardless of the number of eLNs. When fewer lymph nodes are removed, the N classification may be biased, leading to staging migration, inadequate treatment, and impaired predictive accuracy [24]. The current study confirmed that N staging did not adequately discriminate between patients with N0 and N1 disease and between patients with N2 and N3 disease. Increasing the number of eLNs suggested by previous studies might be useful but inevitably leads to overtreatment [4, 6, 12]. Thus, other metrics need to be developed as corrections or alternatives. Second, even at the same TNM stage, prognostic heterogeneity exists among patients because of their age, histologic grade, etc [25, 26]. Due to the specificity of breast IMPC, identifying and optimizing prognostic risk factors and developing instructive novel surveillance systems are crucial.

The LODDS may be the best indicator for assisting the N status in preventing breast IMPC migration and improving prediction accuracy. It considers both positive and negative lymph node status and has been demonstrated to be clinically valuable by many studies [14–17, 21, 25, 26]. Several studies have suggested using pLN and pLNR [27, 28]. However, the pLN is also directly limited by the eLN, and additional help is needed to avoid staging migration [29–31]. Although the pLNR avoided heterogeneity within the same N status, several limitations still restricted its use: 1) if the pLN was 0, the pLNR was always 0%, regardless of whether the eLN was 10 or 20; and 2) if all the eLNs were positive (i.e., pLN =eLN), the pLNR rate was always 100%, regardless of whether the eLNs were 1 or 10. In brief, pLNR failed to risk stratify these patients satisfactorily [30]. These limitations led to the use of the LODDS, which was developed based on the ratio of pLN to nLN, thus eliminating the restrictions of the pLNR. As shown in Fig. 2C, even if a patient had a pLNR of 0% (N0 status), these patients were still better differentiated according to the LODDS. In addition, because the nLN was considered, heterogeneity between patients could be differentiated by the LODDS even if the same pLN profile was present (Fig. 2B). Notably, when the ratio of pLN to rLN was the same (for which the LODDS was always 0), LODDS may also underestimate the lymph node status. Therefore, we considered the LODDS to be a correction rather than a replacement for N staging, and these two criteria worked together to improve the accuracy of the lymph node status of patients with breast IMPC.

The LODDS of breast IMPC has not been mentioned, so its optimal critical value remains undefined. In this study, we analyzed the variation in the survival risk of patients with LODDS for the first time. This was a non-linear relationship: a change in LODDS values within a certain range (LODDS ≤ -1.08) was protective for patients with breast IMPC, and beyond the critical value, this protection was lost, confirming the heterogeneity of the lymph node status. For clinical interpretation and application, we stratified the patients based on this value, and the two subgroups exhibited significant differences in survival. Moreover, multivariate Cox regression analysis revealed that the LODDS score, based on this critical value, was an independent influencing factor of BCSS in IMPC patients. These results suggested that the LODDS had good clinical agreement and deserves further application.

The nomogram was developed as an excellent visualization of the statistical model capable of integrating multiple predictors. As previously discussed, an attempt was made to address the issue of staging migration for N status by employing the LODDS), alongside recognizing the need for additional valuable predictors to enhance the estimation of individualized prognoses [18, 19]. Therefore, predictors were selected through the application of the Cox-LASSO combined with multivariate Cox regression, drawing on their established predictive value in prior research. Among these predictors, tumor size and N status were integral to the traditional TNM staging. Numerous studies have highlighted that patients with breast IMPC often exhibit hormone receptor positivity, which is associated with improved prognosis, likely due to the benefits of long-term endocrine therapy [1, 3, 10]. A high histological grade, indicative of breast IMPC's unique pathological structure, was another significant factor identified [1, 3, 4, 6, 8, 12].

Our study faced several limitations. Firstly, retrospective bias was inherent in the data utilized for this research, necessitating further external validation with prospective datasets to confirm our conclusions. Secondly, while the SEER database provided a comprehensive dataset, it lacked information on certain variables, such as lymphovascular invasion (LVI) status and the Ki-67 index, which could have influenced the study's outcomes. Thirdly, our model gave precedence to tumor size over T stage, a decision influenced by the scarcity of studies linking IMPC with invasions of the chest wall or skin. Additionally, tumor size data were more readily available and easier to apply, potentially limiting the model's applicability to patients with T4 tumors. Lastly, as treatment strategies evolve, the prognosis for breast IMPC patients is expected to change, indicating a need for ongoing refinement and expansion of our model, including the integration of additional prognostic markers, to maintain its accuracy.

## Conclusion

The LODDS was established as an independent prognostic factor for breast IMPC, highlighting the importance of keeping it below –1.08. This metric's value was not compromised by the number of lymph nodes dissected; thus, improving the predictive precision of N status. A nomogram that includes LODDS, N status, tumor size, ER status, and histologic grade was developed. This model effectively quantified risk and demonstrated satisfactory performance, offering the possibility for more accurate, personalized guidance for patients with breast IMPC. Nonetheless, its application still requires validation through prospective studies.

### Supplementary Information

**Supplementary Material 1.**

**Supplementary Material 2.**

## Data Availability

The data used in this study were obtained from the Surveillance, Epidemiology, and End Results (SEER) database (https://seer.cancer.gov/) and were available through the SEER∗Stat software.

## Abbreviations

AIC: Akaike information criterion
AJCC: The American Joint Committee on Cancer
AUC: Area under the curve
BCSS: Breast cancer-specific survival
CI: Confidence interval
C-index: Concordance index
DCA: Decision curve analysis
eLN: Excised lymph node
ER: Estrogen receptor
Her-2: Human epidermal growth factor receptor-2
HR: Hazard ratio
ICD-O-3: International Classification of Diseases for Oncology
IDC: Invasive ductal carcinoma
IDI: Integrated discrimination improvement
IMPC: Invasive micropapillary carcinoma
LASSO: Least absolute shrinkage and selection operator
LN: Lymph node
LODDS: Log odds of positive lymph nodes
nLN: Negative lymph node
NRI: Net reclassification improvement
pLN: Positive lymph node
pLNR: Positive lymph node ratio
PR: Progesterone receptor
RCS: Restricted cubic spline
SEER: Surveillance, Epidemiology, and End Results
TNM: Tumor-node-metastasis
