# Supplementary material for: Log odds of positive lymph nodes (LODDS)-based novel nomogram for survival estimation in patients with invasive micropapillary carcinoma of the breast
Source: BMC Med Res Methodol. 2024 Apr 18;24:90. doi: 10.1186/s12874-024-02218-1 (PMC11025266; doi:10.1186/s12874-024-02218-1)
Supplement: Supplementary file 1 — Supplementary Material 1. [file 12874_2024_2218_MOESM1_ESM.docx]

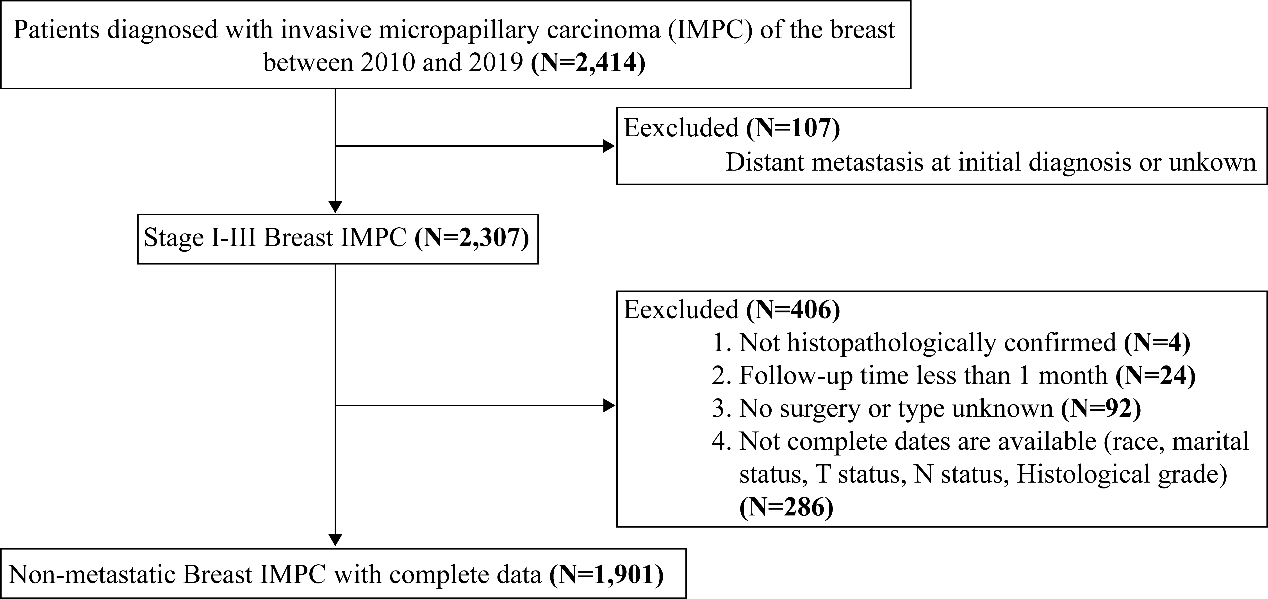


**Additional file 1.** Screening process for patients with invasive micropapillary carcinoma of the breast (IMPC) in the study
