# Supplementary material for: Log odds of positive lymph nodes (LODDS)-based novel nomogram for survival estimation in patients with invasive micropapillary carcinoma of the breast
Source: BMC Med Res Methodol. 2024 Apr 18;24:90. doi: 10.1186/s12874-024-02218-1 (PMC11025266; doi:10.1186/s12874-024-02218-1)
Supplement: Supplementary file 2 — Supplementary Material 2. [file 12874_2024_2218_MOESM2_ESM.docx]

**Additional file 2.** Model comparison

|  | LODDS-based Nomogram | Nomogram without LODDS | AJCC TNM staging |
| --- | --- | --- | --- |
| C-index | 0.802 (95% CI: 0.741-0.863) | 0.782 (95% CI: 0.719-0.845) | 0.692 (95% CI: 0.627-0.757) |
| AIC | 905.169 | 910.066 | 958.694 |
| 5-year |  |  |  |
| AUC | 0.800 (95% CI: 0.728-0.872) | 0.748 (95% CI: 0.668-0.828) | 0.672 (95% CI: 0.592-0.753) |
| NRI | Reference | -19.8% (95% CI: -27.1%~-4.0%), P<0.001 | -19.9% (95% CI: -33.7%~-3.9%), P<0.001 |
| IDI | Reference | -2.8% (-4.7%~-0.4%), P<0.001 | -4.6% (95% CI: -8.1%~-0.6%), P<0.001 |
| 8-year |  |  |  |
| AUC | 0.780 (95% CI: 0.706-0.853) | 0.768 (95% CI: 0.696-0.841) | 0.664 (95% CI: 0.589-0.740) |
| NRI | Reference | -10.0% (95% CI: -22.8%~-2.6%), P<0.001 | -15.2% (95% CI: -25.6%~-4.0%), P<0.001 |
| IDI | Reference | -2.4% (95% CI: -4.9%~-0.3%), P<0.001 | -5.2% (95% CI: -7.7%~-0.5%), P<0.001 |

**Abbreviations:** LODDS, the log odds of positive lymph nodes; AJCC, the American Joint Committee on Cancer, TNM: tumor-node-metastasis; C-index, concordance index; AIC, Akaike information criterion; AUC, the area under the curve; NRI, net reclassification improvement; IDI, integrated discrimination improvement.
